# Biochemistry strategies for label-free optical sensor biofunctionalization: advances towards real applicability

**DOI:** 10.1007/s00216-021-03751-4

**Published:** 2021-11-04

**Authors:** Maria Soler, Laura M. Lechuga

**Affiliations:** Nanobiosensors and Bioanalytical Applications Group (NanoB2A), Catalan Institute of Nanoscience and Nanotechnology (ICN2), CSIC, BIST, and CIBER-BBN, Bellaterra, 08193 Barcelona, Spain

**Keywords:** Surface plasmon resonance, Silicon photonics, Antibody immobilization, Biochemical cross-linking, Antifouling coating, Lipid membrane

## Abstract

Label-free biosensors, and especially those based on optical transducers like plasmonic or silicon photonic systems, have positioned themselves as potential alternatives for rapid and highly sensitive clinical diagnostics, on-site environmental monitoring, and for quality control in foods or other industrial applications, among others. However, most of the biosensor technology has not yet been transferred and implemented in commercial products. Among the several causes behind that, a major challenge is the lack of standardized protocols for sensor biofunctionalization. In this review, we summarize the most common methodologies for sensor surface chemical modification and bioreceptor immobilization, discussing their advantages and limitations in terms of analytical sensitivity and selectivity, reproducibility, and versatility. Special focus is placed on the suggestions of innovative strategies towards antifouling and biomimetic functional coatings to boost the applicability and reliability of optical biosensors in clinics and biomedicine. Finally, a brief overview of research directions in the area of device integration, automation, and multiplexing will give a glimpse of the future perspectives for label-free optical biosensors.

## Introduction

**Fig. 1:**
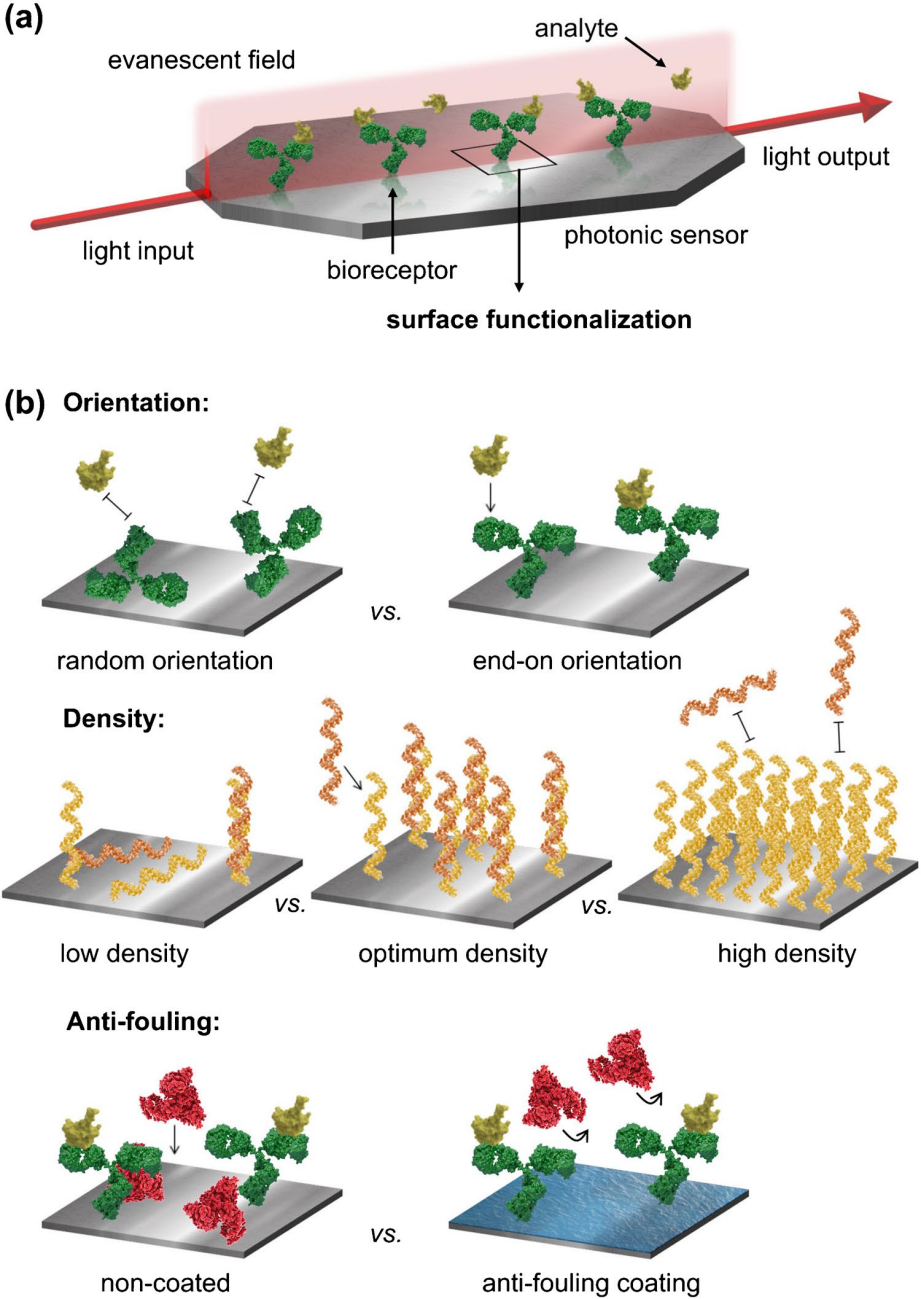
**a** Illustrative scheme of an optical biosensor system based on the evanescent field sensing mechanism: light is coupled to the transducer surface generating an electromagnetic field that penetrates evanescently into the dielectric medium where the biological interaction takes place. The biointeraction changes the refractive index of the medium, which is translated in variations of certain optical properties of the output light (intensity, wavelength, etc.). **b** Main optimization parameters in sensor surface biofunctionalization: bioreceptor orientation (top), grafting density (middle), and antifouling coating (bottom)

Label-free optical biosensors are devices able to detect, quantify, and monitor the presence of analytes of interest with high sensitivity and specificity in just a few minutes of assay directly performed at the point of need [1, 2]. Several optical biosensor nanotechnologies, either based on nanoplasmonics (i.e., surface plasmon resonance (SPR) biosensors) or silicon nanophotonics (e.g., ring resonators, interferometers), have been developed and even commercialized [3–6]. All these sensors rely on the same physical working mechanism, the evanescent field sensing principle [7, 8]. To explain briefly, the evanescent field is an electromagnetic field generated at the interface between a surface where light propagates (i.e., plasmonic surface or waveguide) and a dielectric media (Fig. 1a). It penetrates into the dielectric media with an exponentially decaying intensity, reaching up to several hundreds of nanometers (100–1000 nm). The evanescent field is extremely sensitive to minute changes of refractive index occurring in the dielectric, such as those caused by a change of composition or change of mass on the surface of plasmonic or waveguide-based sensors. The refractive index changes induce a variation in the light propagation parameters, which can be readily measured through spectral peaks displacements, reflectance changes, or phase variations, among others. Thereby, the evanescent field can serve as a probe to detect and analyze biomolecular interactions taking place onto the sensor surface, being monitored in real time and without the need for external tags or labels (e.g., fluorescent or colorimetric tags) and providing a quantitative analysis. These capabilities have positioned label-free optical biosensors as potential technologies for point-of-care (POC) testing, with appealing applications in in vitro medical diagnosis, personalized therapy, or environmental control (safety, pollution monitoring, etc.) [9–12]. In fact, over the last few years, a significant number of publications are reporting label-free optical biosensors for early diagnosis of cancer and other diseases, rapid detection of infectious bacteria or viruses, analysis and monitoring of biomarkers or drug levels in patients during therapy, etc. [4, 13]. The performance of these biosensors often proves excellent when compared to standard clinical diagnostics (i.e., enzyme-linked immunosorbent assays, ELISA, or polymerase chain reaction tests, PCR) in small validation studies with patient samples. But still, the adoption of biosensors in clinical or environmental settings has not been accomplished. Among the several limitations, such as system automation or signal interpretation, an important challenge is the sensor biofunctionalization, which refers to the preparation and chemical modification of the sensor surface for attaching the specific bioreceptor and minimizing non-specific adsorptions [7, 14].

The label-free analysis scheme of optical biosensors imposes critical requirements to the biorecognition interface, as main responsible for the reliability and accuracy of the assay (Fig. 1b). Two aspects must be carefully studied and optimized: the selection of the biorecognition element, which provides the affinity and specificity for the target analyte; and its immobilization on the sensor surface. The bioreceptor immobilization strategy must ensure (i) a uniform coverage and proper biomolecule orientation, to maximize target accessibility and detectability; (ii) stability and robustness, allowing flow-through assays or sequential measurement cycles; and (iii) antifouling capabilities, to avoid or minimize the undesired binding of sample matrix components (e.g., proteins, lipids) to the sensor surface, which can generate false positive signals. In this review, we provide an overview of the variety of sensor biorecognition interfaces developed and demonstrated in label-free optical biosensor technologies. After a brief analysis on the selection of the biorecognition element, we describe in detail the most successful and widely used surface chemistry and cross-linking procedures as well as innovative methodologies recently proposed to extend the applicability of biosensors, to improve its performance, or to boost the technology transfer, commercialization, and implementation in the clinical practice. Finally, current and future perspectives in this area are critically discussed based on recent advances in nanotechnology, materials science, and bioengineering.

## Selection of the biorecognition element

The most common and widely employed bioreceptors are antibodies. Antibodies can be produced towards virtually any type of molecule, including proteins, peptides, small organic molecules like drugs, and even large intact cells (e.g., bacteria). Over the years, the antibody production process has been greatly optimized and improved, ensuring reliable and reproducible collections of high-affinity monoclonal antibodies but also generation of novel recombinant chimeric antibodies [15] or nanobodies [16], which can be easily obtained at large scales with cost-effective procedures. To face certain limitations of antibodies, such as long-term stability, other recognition molecules have been proposed as alternative. It is the case, for example, of molecularly imprinted polymers (MIPs) or aptamers. MIPs are synthetic polymers that are templated during production to selectively bind the target molecule by a “lock and key” mechanism [17]. They exhibit extreme robustness and can be obtained at a low cost, making them especially attractive for biosensors employed at remote locations or under harsh conditions, but their performance in terms of affinity and specificity usually is not sufficient for clinical or biomedical applications [18]. In recent years, a novel approach for nanosized MIPs solid-phase synthesis has been proposed, showing superior affinities [19]. Although mainly applied in electrochemical sensors, nanoMIPs could become a potential alternative to antibodies also in optical biosensors [20]. Aptamers, on the other hand, are single-stranded oligonucleotides (DNA or RNA) with molecular recognition capabilities based on structural folding [21]. They can be chemically synthesized with the desired sequence and functionalities, therefore avoiding animal immunization and cell culture procedures, and their affinity and specificity towards target analyte can be equivalent to monoclonal antibodies. However, the design and selection process for aptamer production can be long and tedious (i.e., SELEX procedure), and often results unsatisfactory for many types of molecules, especially small organic molecules [22, 23].

On the other hand, genomic biomarkers such as micro-RNAs have arisen as promising indicators for early and highly accurate diagnostics. The detection of single-stranded nucleic acid molecules can be straightforward via direct hybridization assays, employing synthetic DNA probes with the complementary sequence. Like aptamers, these DNA probes can be chemically synthesized and purified at relatively large scales and also can incorporate specific functionalities [24]. When addressing complex applications in genomic-based diagnostics, such as the analysis of epigenetic DNA markers or specific DNA mutations, it might be necessary to capture double-stranded DNA. For that, a few bioengineering approaches have been proposed like the formation of triple helices (i.e., triplex) using hairpin-shape DNA strands, which can be easily designed with bioinformatic tools and chemically synthesized [24–26]. Finally, it is worth mentioning that cell receptors can also be employed as biorecognition elements, particularly useful when targeting whole intact cells, like tumor cells or viruses, for example. These receptors are commonly produced as recombinant proteins and have been applied in biosensors for studying cell–cell or cell-pathogen interactions [27]. Table 1 summarizes the advantages and limitations of the main biorecognition elements commonly employed in label-free optical biosensors.

**Table 1:** Advantages and limitations of common bioreceptors used in label-free optical biosensors

Receptor	Advantages	Limitations
Antibodies	• High affinity and specificity • Well-established production process • Wide range of targets: protein, peptide, small molecule, cells, etc	• Long and complex production via animal immunization • High production costs • Activity loss in long-term storage • Low reusability
Bioengineered antibodies	• High affinity and specificity • Wide range of targets: protein, peptide, small molecule, cells, etc • No animal immunization required • Scalable and cost-effective production	• Need to know the sequence, or find binding regions through phage display • Storage and reusability similar to conventional antibodies
Cell receptors	• High affinity and specificity • In vitro production	• Sequence knowledge required • Storage and reusability similar or lower than antibodies • Limited range of targets
Molecularly imprinted polymers	• High robustness and stability • Scalable and cost-effective production	• Moderate-low affinity and specificity • Limited range of targets
DNA strands	• High affinity and specificity • High stability and reusability • Versatile production via chemical synthesis	• High production costs • Limited range of targets • Limited length subject to structural conformations
Aptamers	• High-moderate affinity and specificity • Production via chemical synthesis • Stability and long-term storage • Reusability	• Long and complex selection procedure via SELEX • Moderate range of targets • Subject to structural conformations

## Bioreceptor immobilization strategies

The methodology for bioreceptor immobilization depends on the type of biorecognition element (i.e., protein, nucleic acid, etc.) and its functionality, but also on the target analyte (e.g., nature, dimensions), since it will eventually determine parameters such as the optimal density that minimizes steric hindrance effects during the interaction. Given the broad variety of biorecognition elements that can be used for biosensing, here we focus on the two main and most widely employed types: antibodies and oligonucleotides (i.e., single-stranded DNA probes and aptamers). In the following sections, we will present and discuss a wide variety of approaches to immobilize these bioreceptors onto sensor surfaces, analyzing their advantages and limitations in terms of simplicity and efficiency, uniformity and orientation, versatility, and robustness.

### Physical and chemical adsorption

The simplest approach for sensor biofunctionalization is the physical adsorption, in which bioreceptors are attached directly to the sensor surface by electrostatic and hydrophobic forces. This scheme is employed for microwell plate functionalization in ELISA, for example. But despite being rapid and straightforward, physisorption can lead to serious reproducibility and stability problems in biosensors, and importantly, it may affect the recognition activity of the bioreceptor molecules, especially for antibodies that can be denaturalized in direct contact with metallic surfaces [28]. Another easy and widely used strategy is the direct chemisorption of bioreceptors, especially those carrying a thiol-functional group (e.g., thiolated DNA probes) that can directly bind to gold sensor surfaces. This procedure is efficient and rapid, resulting in a stable receptor layer. To ensure layer uniformity and bioreceptor orientation, oligonucleotide probes can be synthesized incorporating a vertical spacer between the recognition sequence and the functional group, which can be either a 6- or 12-carbon chain [29], or a poly-Thymine (polyT) sequence (e.g., polyT_10_ or polyT_15_) [30]. The vertical spacer aids in the upright positioning of the probe especially in gold-based sensors thanks to its low affinity for metallic surfaces, but it should be avoided when analyzing samples containing RNA polyA tails, which could bind to the polyT sequence and give rise to false positive signals [31]. For the case of antibodies or other protein receptors, terminal Cysteine residues with reactive thiol groups could be employed for direct chemisorption on gold surfaces. Otherwise, an interesting strategy is to perform antibody fragmentation by enzyme digestion or chemical reduction, releasing sulfhydryl reactive groups that can be attached to the surface [32, 33]. In all these approaches, adjustment of the bioreceptor grafting density is commonly done by adding competing thiolated molecules (e.g., mercaptohexanol, MCH) to act as lateral spacers. This direct competition however results in a relatively low control on the receptor grafting density, as their interaction with gold may be influenced by not only the initial concentration, but the molecular size, electrostatic interactions, etc. An interesting approach has been recently proposed in which the bioreceptor (antibody or DNA probe) is linked to a polyAdenine (polyA) tail, which can be directly adsorbed on gold surfaces by high-affinity electrostatic interactions, and due to its flat positioning can act also as lateral spacer [34, 35]. The polyA-based strategy has been demonstrated and evaluated in a SPR biosensor, showing comparable results to other widely used methodologies, both in terms of analytical sensitivity and selectivity.

### Chemical cross-linking to functional monolayers

The sensor biofunctionalization procedures that have shown the best results in terms of robustness and versatility are those based on the formation of functional chemical monolayers. The sensor surface is chemically modified to provide a homogeneous coating that extends up to 1 or 2 nm from the sensor surface, with terminal functional groups that can be used to tether the bioreceptors via covalent cross-linking or through high-affinity molecular systems. In this way, biorecognition elements are firmly attached to the sensor surface in a controlled and reproducible manner, can be slightly distanced from the surface, and it also allows to coat the entire sensor surface for preventing non-specific adsorptions.

**Fig. 2:**
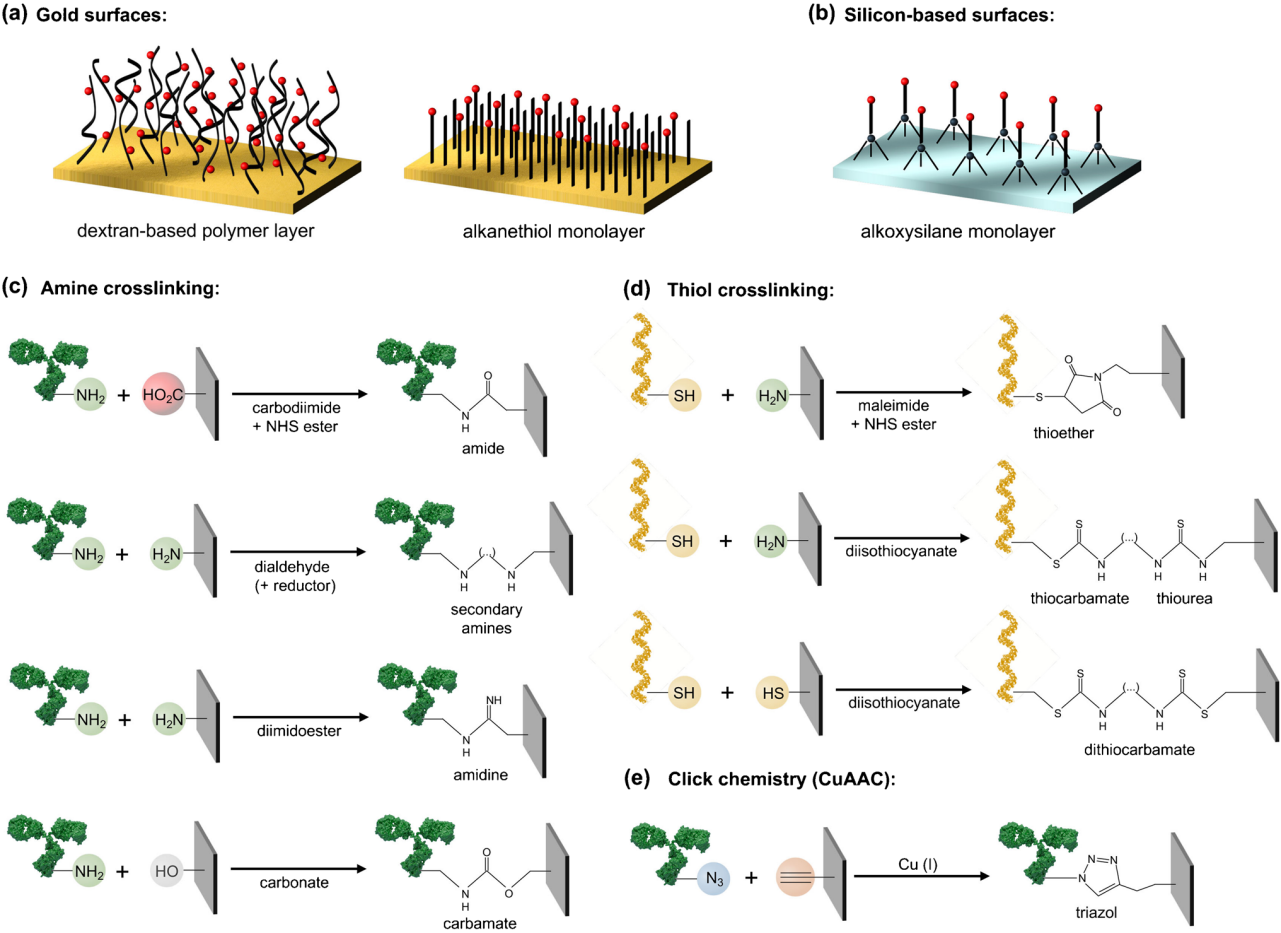
**a** Schematics of chemical scaffolds for gold-based sensor functionalization: dextran-based polymeric layer (e.g., carboxymethyl dextran) (left) and mixed alkanethiol self-assembled monolayer (e.g., mercaptohexadecanoic acid/mercaptoundecanol, MHDA/MUOH) (right). **b** Schematics of chemical scaffold for silicon-based sensor functionalization: alkoxysilane monolayer (e.g., 3-aminopropyl(triethoxysilane), APTES). **c** Schematics of different examples of cross-linking strategies for amine-functional bioreceptors. **d** Schematics of different examples of cross-linking strategies for thiol-functional bioreceptors. **e** Schematics of cross-linking strategy based on click chemistry: copper(I)-catalyzed alkyne-azyde cycloaddition (CuAAC). Antibodies and DNA strands are only used for illustrative purposes; any receptor carrying the desired functionality could be employed indistinctly

For gold plasmonic sensors, two major functional chemical scaffolds are used: dextran-based polymers and alkanethiol self-assembled monolayers (SAMs) (Fig. 2a). Functional polysaccharides, such as carboxymethylated dextran, are known to form highly stable, compact, and hydrophilic layers on gold surfaces, containing reactive groups (e.g., COOH) that can be used for bioreceptor coupling [36]. This approach has become very popular especially for SPR biosensors, with dextran-modified sensor chips commercially available (CM5 sensors, Biacore, GE). However, the random distribution of functional groups within the polymer layer might hamper the optimum and efficient cross-linking for bioreceptor binding. On the other hand, SAMs are formed by chemisorption of short-chain organic alkanethiols, i.e., carbon chains with terminal sulfur-reactive groups (-SH) [37, 38]. These molecules generate a densely packed and highly ordered hydrophilic layer on the sensor surface, displaying functional reactive groups at the outer end of the scaffold, therefore being available for bioreceptor cross-linking. A myriad of different alkanethiols is commercially available, being relatively easy to synthesize them incorporating different chemical end functionalities, such as carboxyl (COOH), amine (NH_2_), and hydroxyl (OH). Furthermore, SAMs can be formed as a mixture of different alkanethiols varying the reactive/inert group ratios of the monolayer, which offers the possibility to easily control and adjust the bioreceptor grafting density according to the target dimensions, minimizing steric hindrance issues.

In silicon-based photonic biosensors, the most common materials for sensor waveguide fabrication are either silicon oxide (SiO_2_) or silicon nitride (Si_3_N_4_) and chemical modification of these surfaces is generally done via silanization (Fig. 2b) [39]. Functional alkoxysilanes can form ordered and stable monolayers in silicon-derived substrates similar to alkanethiol SAMs. However, silanization is a more complex procedure than thiol chemisorption and there is currently no consensus on the optimum parameters to achieve a stable and homogeneous monolayer. The silanization process essentially consists in three main steps: surface activation, to release reactive hydroxyl groups (-OH) on the substrate; silane binding, which can be done in liquid or vapor phase; and finally, a curing step to cross-link the silane molecules and stabilize the monolayer. For this procedure, numerous protocols have been reported with different reaction times, temperatures, solvents, and even atmospheric conditions. The most common protocols employ amino-functional silanes, such as the (3-aminopropyl)triethoxysilane (APTES), which provide stable films through, for instance, vapor deposition at room temperature for 4 h [40], or vapor deposition at 150 °C for 5 min [41], or liquid deposition in anhydrous toluene [42]. These examples suggest that optimum silanization can either be achieved in different ways or it needs to be particularly and carefully studied for each specific material of the sensor surface. Moreover, as in the case of alkanethiols, different functional silanes can be employed for sensor surface modification (e.g., COOH-terminated silanes), and silanization procedure might be adjusted for each case.

Once the functional scaffold is formed on the sensor surface, the subsequent step is the anchoring of the bioreceptor molecules, which is the most delicate and critical process to achieve the highest detection sensitivity and accuracy. To achieve a highly stable and robust bioreceptor tethering on the functional layer, the primary strategy is to chemically link their reactive functional groups forming a covalent bond (Fig. 2c, d, e). This reaction generally requires the activation of certain groups and/or the use of cross-linkers. The carboxyl-amine cross-linking is probably the most well-established strategy. Generally, this reaction is carried out through a well-known carbodiimide-based chemistry, employing a mixture of EDC (1-ethyl-3-(3-dimethylaminopropyl)carbodiimide) and NHS (N-hydroxysuccinimide) as intermediate cross-linkers, and resulting in a highly stable amide bond between the biomolecule and the scaffold [43]. This reaction provides very good yields and good surface coverage with a high reproducibility. Another conventional approach is the amine–amine binding, for which several cross-linking methods can be employed [44, 45]. The simplest is the use of glutaraldehyde, a small molecule with highly reactive carbonyl groups (-CHO) that condense primary amines very efficiently via reductive amination or Mannich reactions. Homobifunctional NHS ester cross-linkers, like disuccinimidyl suberate (DSS) or bis(sulfosuccinimidyl) suberate (BS3), are however the most popular ones, offering a better control on the cross-linking reaction as well as the incorporation of a spacer arm that can be customized in length. Other cross-linking strategies employed for primary amine coupling are based on epoxides, isocyanates, or imidoesters, among others, that results in either amide or amidine bonds, but with certain complexity in terms of side products, reaction time and conditions, etc. More sophisticated cross-linking reactions have also been studied and employed, like the click chemistry-based methods. Although several types of click reactions have been described, the most common one is the Cu-catalyzed azide-alkyne cycloaddition (CuAAC) that employs Cu^I^ catalysis to efficiently couple azide groups to terminal alkynes (Fig. 2e) [46]. The advantages of click chemistry are its chemoselectivity, high yields, and no production of side products; however, it usually requires previous modification of the probe with an azide-functional tag.

The versatility and robustness of covalent binding strategies have made them one of the most widely employed approaches in antibody immobilization for biosensing. However, it does not offer any control over the orientation of the biomolecules, which is mandatory to maximize analyte detection capability. It is also important to carefully optimize the reaction parameters (e.g., pH, ionic strength, temperature) to ensure maximum yields and efficiency, avoiding possible electrostatic repulsions between the monolayer and the bioreceptor.

### Affinity and linker-mediated immobilization

**Fig. 3:**
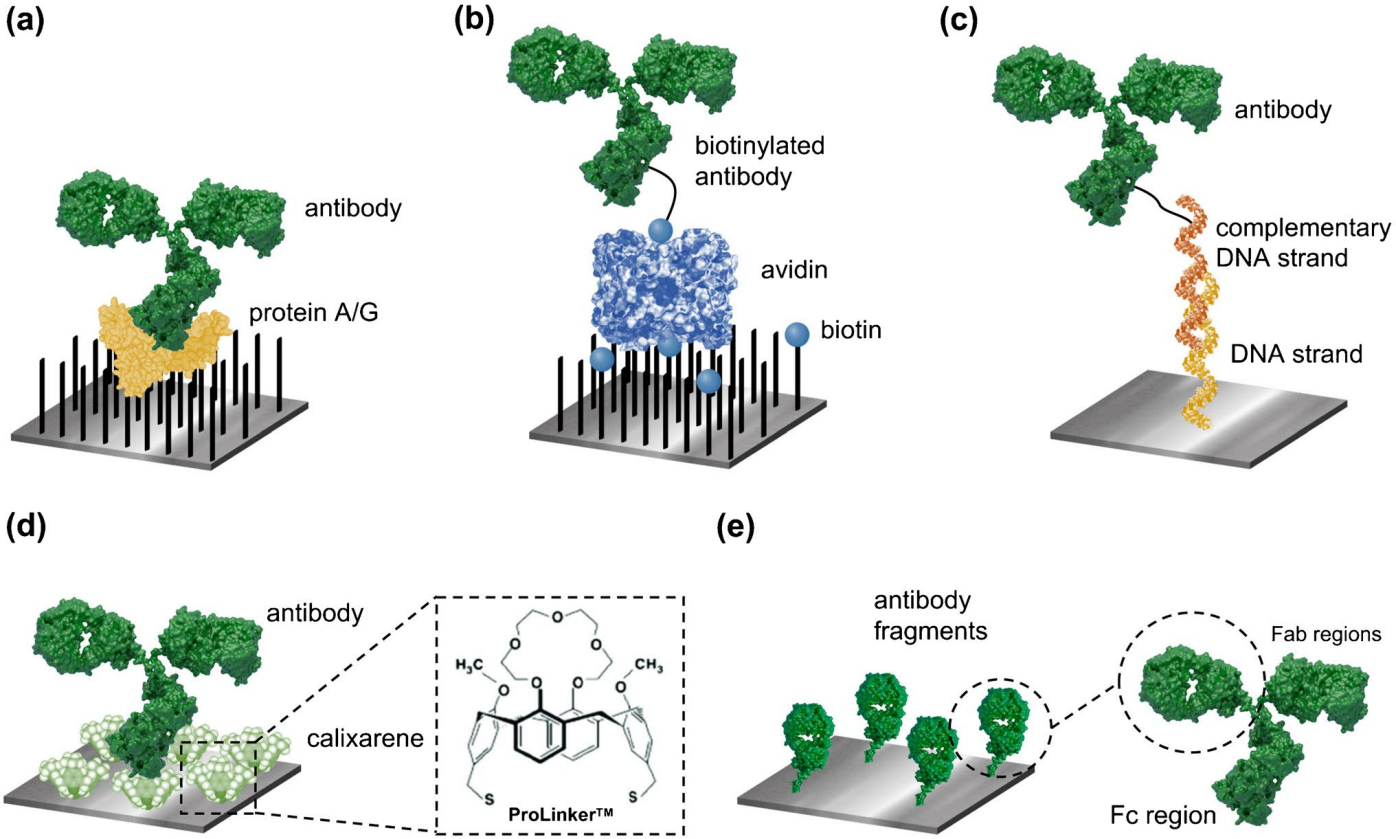
Antibody orientation strategies. **a** Affinity-based immobilization of intact antibodies on Protein A/G. **b** Affinity-based immobilization of biotinylated antibodies on avidin protein (e.g., streptavidin or neutravidin). **c** Affinity-based immobilization through DNA hybridization. **d** Immobilization of intact antibodies on a calixarene-based linker (e.g., Prolinker™). **e** Immobilization of recombinant antibody fragments, i.e., antigen-binding region (Fab)

A proper orientation of the bioreceptors is essential for exposing the binding sites towards the analyte, and for achieving a uniformly distributed bioreceptor layer, which would ensure higher recognition efficiency, selectivity, and reproducibility. This is especially relevant for antibodies, which cannot be custom synthesized and therefore require certain biochemical strategies to be immobilized in a suitable orientation (Fig. 3). The classic example is the Protein A or Protein G approach [47]. These two proteins—and the recombinant combination of both (Protein A/G)—are able to selectively capture different types of immunoglobulins through their constant region without the need for chemical manipulation of the antibodies. The affinity protein can be covalently linked to the sensor surface monolayer, and antibodies are captured on them, resulting in a sterically accessible and perfectly orientated recognition interface (Fig. 3a). However, the interaction between protein A/G and antibodies is not particularly strong and can be disrupted with a change of pH, which limits the stability of the biolayer and the use of the biosensors for sequential measurement cycles. Another common strategy employs the biotin-avidin system. These two molecules showed one of the highest affinity interactions known in biology (*k*_d_ ≈ 10^−14^ mol/L); thereby, the binding of a biotinylated-Ab to an avidin layer results in a highly stable recognition interface (Fig. 3b) [48]. If antibodies are conjugated to the biotin tag through a site-specific procedure (i.e., through the carbohydrate moieties of the Fc region), the immobilization will also be oriented. Another interesting approach would be the DNA-directed immobilization. It consists in conjugating the antibody to a DNA oligonucleotide strand—ideally through the constant region—and anchor it to the sensor surface via hybridization with the complementary sequence probe, previously immobilized on the sensor surface (Fig. 3c) [49].

These strategies provide oriented and stable recognition interfaces, but as a drawback, they require chemical modification of antibodies for tag conjugation. In this regard, an innovative methodology was reported a few years ago based on calixarenes (e.g., ProLinker™) [50]. Monolayers formed with calixarene crown ethers on the sensor surface can serve as scaffolds to directly immobilize intact antibodies through their Fc region with a high stability and in a simple and rapid procedure (Fig. 3d). Nonetheless, this line has not been further investigated in recent years because the commercial product was discontinued. The design and synthesis of novel similar calixarenes could lead to a promising strategy for oriented antibody immobilization. Finally, another attractive trend is to use genetic bioengineering procedures to directly produce recombinant Fab fragments, which can be tagged with histidine or cysteine residues or fused to larger proteins that aid in the immobilization (Fig. 3e) [51]. This strategy has also been combined with other approaches, like biotin-avidin system or protein-binding formats.

All over, a vast library of bioreceptor immobilization strategies has been proposed, studied, and employed in label-free optical biosensors. In order to achieve maximum functionality, binding capacity, and assay reliability, the attachment procedure must ensure the correct antibody orientation together with an optimum surface coverage and interface stability. Comparative studies of different immobilization strategies reveal that oriented receptor layers provide a higher sensitivity and binding efficiency while random orientation approaches are simpler, faster, and provide higher density of receptors. Eventually, the selection of the most appropriate strategy will depend on the selected application of the biosensor and its performance requirements.

## Towards an antifouling surface

**Fig. 4:**
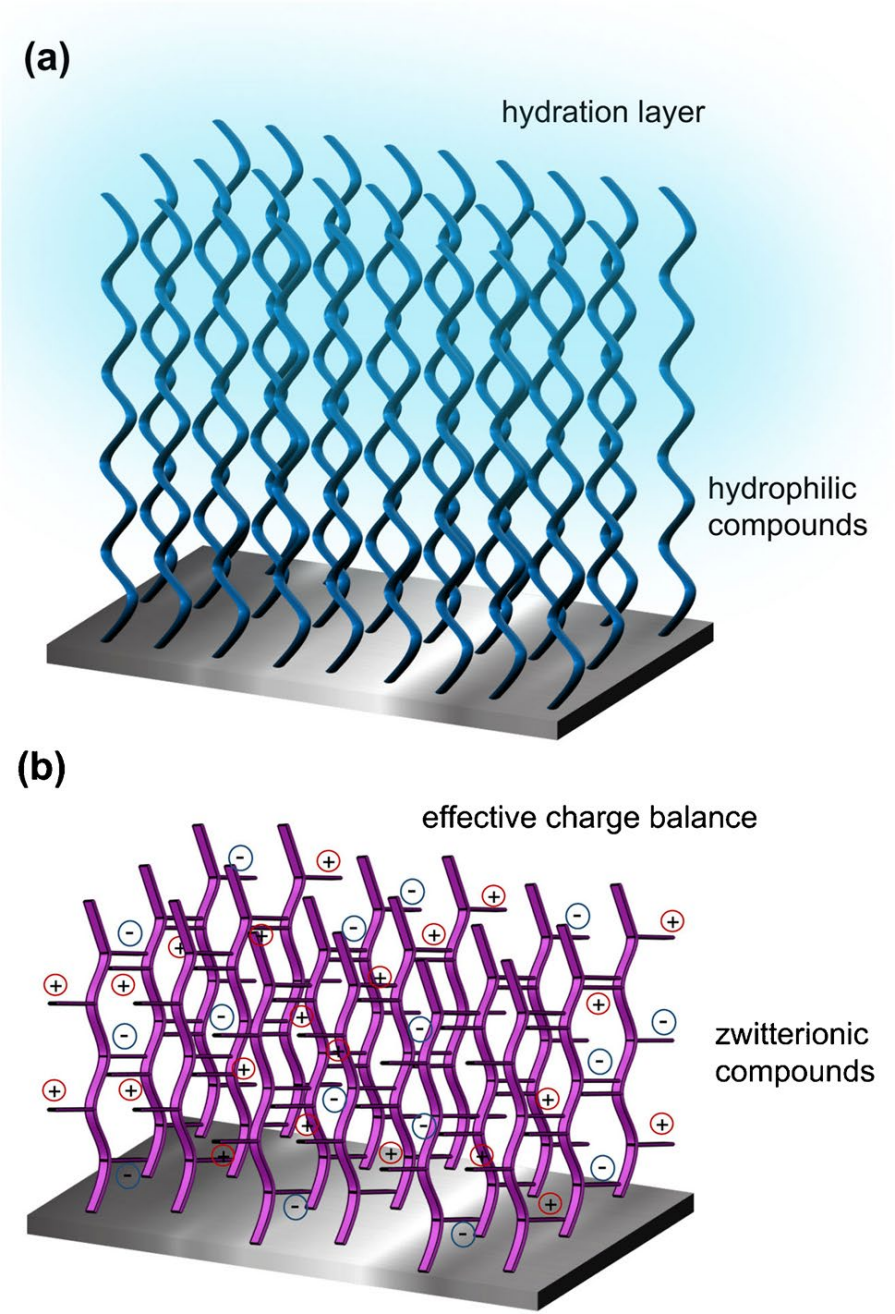
Antifouling strategies. **a** Formation of a hydration layer with hydrophilic compounds, e.g., incorporating polyethylene glycol or oligoethylene glycol moieties. **b** Formation of an effective charge-balanced layer with zwitterionic compounds

Despite all the efforts in accomplishing stable, robust, uniform, and oriented biorecognition interfaces, there is still an unresolved challenge: eliminating non-specific adsorptions from sample matrix components (e.g., proteins, lipids, cells). This undesired adsorption is mainly due to long-range electrostatic and Van der Waals interactions. Most efficient mechanisms to minimize it are directed to further increase the surface hydrophilicity, generating a hydration layer (i.e., organized water molecules layer at the sensor coating), and/or alter the effective surface charges of the chemical scaffold formed on the sensor surface to reduce the ionic interactions (Fig. 4).

A popular approach is to form functional monolayers incorporating oligoethylene glycol (OEG) moieties (Fig. 4a). OEG-terminated SAMs offer a high hydrophilicity and lateral packing density that increases resistance to protein fouling [52, 53], which have been successfully used to minimize adsorption of proteins from blood-related fluids, but insufficient to completely avoid it when dealing with undiluted blood, plasma, or serum samples (Table 2) [54, 58]. It has been shown that the antifouling capacity improves with the number of EG molecules in the SAM; therefore, surface coatings with different polyethylene glycol (PEG) compounds have also been candidates for functional low-fouling scaffolds [54, 55]. Commonly used compounds include linear PEGs, such as silane-PEG-*R* or thiol-PEG-*R* (where *R* is a functional reactive group, e.g., COOH, NH_2_) for silicon and gold surfaces [59, 60], respectively, and also polymer brushes containing PEG chains grafted to a functional backbone, such as poly(L-lysine) (PLL-PEG) [61]. Grafted polymers have proven higher resistance to protein fouling; however, if grafting density and position are not well controlled, these long-chain PEGylated compounds can form disordered scaffolds where the reactive functional group for bioreceptor immobilization becomes hindered [59, 62].

**Table 2:** Adsorption from protein solutions and undiluted blood plasma on different surface coatings

Coating	Fouling (pg/mm^2^)*			Ref
	Fibrinogen	HSA	Blood plasma	
OEG_2_	300	105	1500–2500	[54, 55]
OEG_6_	36	0	500	[54, 55]
PEG-SAM	30	0	900	[55]
pDOPA	0	-	63	[56]
pHEMA	0	0	30–35	[57]
poly(β-peptoid)	0	2	97	[57]

^*^Measurements performed by SPR and/or ellipsometry

Another effective antifouling functionalization strategy is the formation of zwitterionic layers (Fig. 4b). This coating is characterized by containing both cationic and anionic groups that provide an effective charge balance on the surface, reducing adsorptions of proteins through electrostatic interactions [63]. Zwitterionic interfaces can attract water molecules via hydrogen bonds and ionic solvation, thereby more strongly than just hydrophilic compounds. Most common examples of zwitterionic monolayers are terminated in carboxybetaine (CB) or phosphorylcholine (PC) groups [64, 65]. These zwitterionic coatings have shown an excellent reduction of blood-related proteins, but again they failed when challenged to undiluted plasma or other media. Similar to PEG/OEG compounds, the antifouling capacity can be improved grafting zwitterionic polymer brushes, such as the poly(carboxybetaine methacrylate) (pCBMA), which generates functionalizable interfaces with ultralow fouling properties [66–68]. The drawback here is that during the preparation of these polymer brushes, there is a lack of control on the thickness that could extend up to several hundreds of nanometers, distancing the target detection interaction from the sensor, where the evanescent field is most sensitive.

Besides PEG/OEG and zwitterionic compounds, other polymers have also been studied as antifouling scaffolds for biosensors such as polydopamine (pDOPA) [69–71], polyacrylamide [72] or poly-(hydroxyethyl methacrylate) brushes (pHEMA) [73], or the more recently proposed, poly(β-peptoid)s [74, 75]. Peptoids are structural isomers of peptides that differ in the linking position of the functional sidechains, which are located in the α-nitrogen instead of the α-carbon of the backbone. This sidechain rearrangement increases the conformation flexibility and hydration capacity, making them ideal biomimetic compounds to form hydrophilic brushes with elevated fouling resistance. All these strategies have shown very promising results towards total resistance to protein and cell adsorptions (Table 2); however, the bioreceptor cross-linking onto these polymer brushes is not well controlled, which limits the final sensitivity of the biosensor.

More studies in the design of new functional and antifouling surfaces are required to allow the desired application and implementation of optical label-free biosensors for direct and reliable analysis of untreated clinical samples like blood.

Many research works have studied and reported the low-fouling behavior of different materials and surface chemistry strategies, mainly analyzing the adsorption of serum proteins like albumin, fibrinogen, or immunoglobulins, to gold sensors (i.e., SPR). However, in most cases, the fouling resistance properties have resulted insufficient when dealing with whole blood plasma samples, or when comparing different pooled plasma or clinical specimens from different individuals. On the other hand, it has been shown that relatively large coatings and polymer brushes offer the highest fouling resistance but paying little attention to the hindering of bioreceptor coupling or the biointeraction distancing from the sensor surface, which would decrease the overall analytical sensitivity for biosensor application. In view of this, on-going research is directed to develop innovative grafting methods (in-solution or on-chip) that provide a precise control of the layer structure, grafting density, polymer length, etc., and ensure highly stable attachment to the sensor surface [76–78].

## Towards a biomimetic microenvironment

The sensor biofunctionalization approaches described and studied so far have been mostly applied for the detection and quantification of specific molecular targets (i.e., proteins, nucleic acids, pathogens, etc.) with medical or environmental diagnostic purposes, essentially. Label-free optical biosensors, however, can also monitor the biomolecular interactions in real time and in their native forms (i.e., without fluorescent tags), and this capability makes them greatly useful for pharmacokinetic analysis or fundamental biology studies, such as drug-receptor interactions or cell–cell interaction, for example. In this regard, surface bioengineering strategies have been described in order to provide biomimetic scaffolds on the sensor surface that can simulate natural biological systems, in which membrane proteins and receptors can maintain their tertiary structure and flexibility for movement, clustering, etc.

**Fig. 5:**
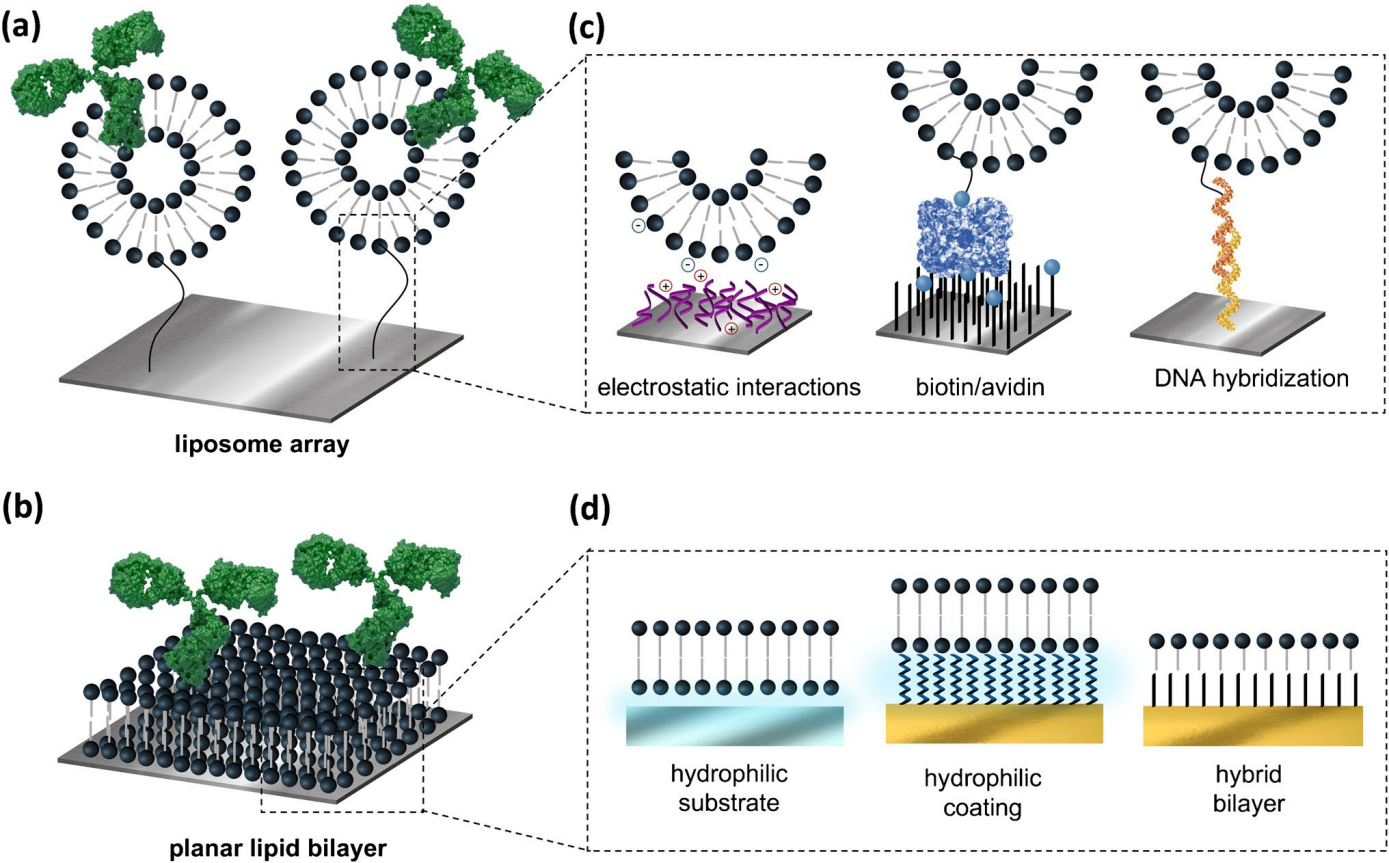
Biomimetic lipid-based biofunctionalization. **a** Schematic illustration of liposome-based bioreceptor immobilization. **b** Schematic illustration of bioreceptor immobilization on a planar lipid bilayer. **c** Representative examples of liposome tethering strategies: electrostatic interactions (left), biotin/avidin system (middle), and DNA-directed (right). **d** Representative examples of planar lipid bilayer formation strategies: supported lipid bilayer (SLB) on hydrophilic substrate (i.e., glass) (left), SLB on hydrophilic coating on gold surface (middle), and hybrid lipid bilayer formed by hydrophobic SAM coating on gold and single lipid layer (right)

Lipid membrane interfaces offer a potential solution to this challenge. Three-dimensional (3D) or bidimensional (2D) lipid-based structures can be deposited, arrayed, or directly grown on sensor surfaces through versatile biochemical engineering procedures starting from relatively low-cost phospholipid molecules, which can be synthesized with the desired properties and functionalities. Mainly, two different lipid-based approaches have been studied for optical sensor biofunctionalization: liposome arrays and planar lipid bilayers (Fig. 5a and b). Liposomes of different sizes can be formed in an aqueous solution by spontaneous self-assembling of phospholipid molecules, like phosphocholine and its derivatives [79]. The hydrophilic head of the phospholipid can be functionalized to incorporate chemical groups, tags, or linkers to facilitate their immobilization onto the sensor surface. The most common strategies for liposome tethering on the sensor surface are based on electrostatic or covalent interactions [80, 81], the biotin-avidin system [82, 83], or by DNA hybridization (Fig. 5c) [84, 85]. The latter is especially interesting for high-throughput multiplexing array sensors; different liposomes can be conjugated to different oligonucleotide sequences that will hybridize to their corresponding complementary sequence previously arrayed on the sensor surface [86]. Major challenges in 3D liposome array formation are closely related to surface antifouling properties and non-specific adsorptions. The sensor surface must be completely passivated before liposome tethering to ensure precise site-selective attachment, to retain the liposome structure, and to avoid the non-specific binding of receptors or targets directly onto the sensor surface [87]. Upon liposome immobilization, the bioreceptor is either inserted or attached to the liposome membrane. As stated before, bioreceptor attachment to the lipid external surface can be done via covalent cross-linking methods or other linker-mediated systems as described previously in “Bioreceptor immobilization strategies.” Nonetheless, when studying drug-cell interactions or ion channel biology, bioreceptors generally are intramembrane proteins that must be inserted in their native conformation. For that, two main approaches can work: using native or synthetic liposomes. Native liposomes can be extracted from cells by techniques as solvent-assistant extraction, sonication, extrusion, freeze/thaw processes, etc. [88, 89]. These native liposomes would exactly mimic the native cell environment, in terms of fluidity and lipid order, but they offer poor control on the amount and type of receptors, and other membrane proteins and components, that can be eventually immobilized on the sensor surface. Conveniently, synthetic liposomes can be formed by directly mixing the bioreceptor molecule with the phospholipid mixture before rehydration [90]. The use of purified compounds eliminates possible interfering molecules (e.g., other membrane proteins) as well as to easily modify the number of receptors per liposome according to the sensor performance requirements or assay parameters. Moreover, liposomes reconstituted from pure components are most likely to have a higher stability for in vitro studies than native liposomes, which could be degraded by enzymes during preparation.

As an alternative to liposome arrays, planar lipid membranes can also be assembled on the sensor to mimic cell surfaces. These thin-film coatings formed by phospholipid bilayers are commonly not chemically attached but supported on the sensor surface [91]. Supported lipid bilayers (SBL) are formed mostly by the vesicle fusion method that consists in the spontaneous rupture of small lipid vesicles in contact with the solid surface and subsequent re-assembly in a planar bilayer architecture [92, 93]. A necessary condition for the vesicle rupture and fusion process to happen is to have a highly hydrophilic surface that attracts phospholipid heads and repeals their hydrophobic tails, aiding in the vesicle disruption and bilayer formation (Fig. 5d). Silicon-based sensor surfaces, and especially glass, have been successfully used for SBL formation; however, manifold factors and parameters must be carefully controlled, including lipid properties (e.g., vesicle size, composition, and concentration), surface properties (e.g., morphology, topography, and atomic composition), and environmental properties (e.g., temperature, ionic strength, buffer composition, pH) [94]. Formation of SLB on gold surfaces results in even more complicated, as generally vesicles adsorb on the surface and remain intact mainly due to the loss of hydrophilic behavior. Some strategies have been developed to aid in this purpose, such as the coating of gold sensors with highly hydrophilic material (e.g., PEGylated polymer brushes), or the formation of hybrid bilayers, in which gold is functionalized with a hydrophobic SAM that mimics the phospholipid tail, and vesicles disrupt to form a single layer on top of the SAM (Fig. 5d) [27].

Overall, lipid-based biofunctionalization strategies have been widely studied and a few innovative mechanisms for liposome arraying or planar bilayer formation have been proposed. It is the case, for instance, of liposome networks (i.e., liposome arrays interconnected through lipid nanotubes) [95], solvent-assistant on-chip SLB formation [96], or patterning of SLB patches via microcontact printing [97], among others. Nonetheless, the application of lipid bioengineered surfaces in label-free optical biosensing is still in its infancy and unfortunately far from industrial scalability and potential technology transfer [98].

## Conclusions and future directions

An optimum sensor biofunctionalization could be the key to accelerate the technology transfer and definitive implementation of powerful optical label-free biosensors in clinical and environmental analysis practices. An endless number of surface coatings, cross-linking strategies, and innovative biochemical procedures have been proposed to achieve a fully reliable and reproducible protocol for immobilizing bioreceptors in an oriented and controlled manner, pursuing maximum assay sensitivity and selectivity. Among them, the covalent binding of bioreceptors to functional chemical matrices formed onto the sensor surface is the most popular and versatile approach. Self-assembled monolayers of alkanethiols on gold or alkoxysilanes on silicon-based sensors provide organized scaffolds for biomolecule immobilization and can be easily adapted to modify the grafting density, to add affinity tags or linkers, and to increase fouling resistance. The latter is, in fact, the major challenge for biosensor application in point-of-care testing. It has been shown that generating a hydration layer on the coating through incorporation of hydrophilic moieties (e.g., ethylene glycol) is necessary for reducing non-specific protein adsorptions, but so far insufficient for completely avoiding fouling from undiluted media. Research in the field proposes new alternatives based on polymeric materials and combinations with zwitterionic compounds that alter the effective net charge of the sensor surface to prevent as well undesired electrostatic interactions. However, the large variability found in real samples composition is still a handicap to successfully eliminate sample pretreatment processes, like dilution or filtration. Current perspectives in this area rely on the controlled growth of novel materials with desired chemical composition and distribution of functional groups to accomplish highly ordered scaffolds, with enhanced fouling resistance properties, that ensure an efficient, simple, and reliable immobilization of all types of bioreceptors.

On the other hand, innovation in surface biofunctionalization could also expand the applicability of optical biosensors in pharmaceutics and molecular biology studies. By exploiting the label-free and real-time monitoring capabilities, these systems can greatly aid in the evaluation of novel drugs, the understanding of cell interactions, etc. To that, several strategies based on lipid bilayer coatings have been suggested to create a biomimetic microenvironment onto the sensor surface in which the bioreceptors behave as they are found in native conditions, increasing the accuracy and reliability of the biological assays. These bioengineered surfaces have shown promising results in model studies, but the functionalization procedure is delicate and complex, requiring sophisticated biochemical techniques that hamper the scalability and adoption in routine experiments. Here, the integration of lab-on-a-chip microfluidic systems that facilitate in-situ preparation or process automation would suppose an enormous boost for the demonstration and implementation of novel biomimetic sensors.

It is important to remark that surface chemistry cannot move forward without the accompaniment of technology and materials development. Potential final users of optical label-free biosensors expect automated and user-friendly systems able to detect and quantify several target analytes of interest in a sample, in a few minutes, with maximum sensitivity, and at affordable prices. Sensors should be delivered ready to use, which implies the industrialization of the biofunctionalization procedure plus packaging methods that protect and ensure the stability and biological activity of the coating and immobilized receptors. The multiplexing capability is nowadays thought of as an engineering challenge, but also surface biofunctionalization protocols must be prepared considering an extreme reduction of reaction volumes or the need of incorporating different functional groups for site-selective immobilization of different receptors. Overall, it becomes clear that despite the vast knowledge and the myriad of strategies developed along the last years for sensor biofunctionalization, there is still a long research way ahead in which nanotechnology, biotechnology, and materials sciences might play the leading role.

## Abbreviations

OEG: Oligoethylene glycol
pDOPA: Poly(dopamine)
PEG: Polyethylene glycol
pHEMA: Poly(hydroxyethylmethacrylate)
POC: Point of care
SAM: Self-assembled monolayer
SLB: Supported lipid bilayer
SPR: Surface plasmon resonance
